# Chronic Inversion of the Uterus

**Published:** 1860-03

**Authors:** F. Ramsbotham

**Affiliations:** London


*Chronic Inversion of the Uterus.* By Dr. F. Ramsbotham, *of London.*

The following case is related by Dr. Ramsbotham, of the London Obstetric Hospital. It is interesting as showing the possibility of spontaneous replacement of an inverted uterus, after it had been displaced for some months, and even after some very rough treatment:

"I was sent for on July 20th, 1839, to see a young lady of relaxed fibre and cachectic habit, who had been delivered of her first child, after a severe labor, twelve hours before, and whom I found suffering from violent forcing-pain and great hæmorrhage. She was exceedingly depressed. I detected a tumor, occupying the vagina, as large as a man's closed fist, entirely covered by a layer of coagulum. It was sensitive, though not painfully so, possessed a doughy feel, and became harder when compressed. The uterus could not be felt in the abdomen; but above the pubes there was a sensation of a most unusual void. As she had passed no urine since delivery, I relieved the bladder; and having not doubt that the tumor was the uterus inverted, I made strenuous efforts to restore it, without effect, but not without putting her to considerable pain. After sometime I desisted in despair, fearing that I should lacerate the upper part of the vagina; for the tumer had become hard while I was making these efforts, and in the same proportion, the circle of the mouth became closed around it in the form of a forcibly constricted ring. She passed a quiet night under the influence of morphia, and in the morning voided urine naturally, with some coagula. She was confined in bed for two, and in the house for three months, with severe lumbar pains, and copious irregular hæmorrhage. From the middle of October till the end of December, she was free from flooding, but still annoyed by bearing-down pains, attended by a profuse, glairy, leucorrhœal discharge.—The hæmorrhage returned, and continued for two months, after which she was moved into the country, and I did not see her again till May 22d. She was then in such imminent danger, that, in consultation with Mr. Hamilton, at that time assistant surgeon to the London Hospital, and with her general attendant, it was agreed to remove the tumor by ligature as soon as she was recruited. It was at this time the size of a ordinary nonpariel apple, and had every characteristic of an inverted uterus.

On June 5th, with the assistance of the gentlemen above mentioned, I placed a ligature around its upper part by means of the double canula. The application gave but little pain at the moment, and the bleeding, which had been going on almost uninterruptedly, ceased immediately. In three or four hours, hours, however, a violent rigor supervened; this was followed by symptoms of intense peritoneal inflammation, and the ligature was removed twenty hours after its application. The pain and other inflammatory symptoms gradually subsided; in a few days she was able to leave her room; she menstruated on July 13th, less profusely than she had been accustomed to do, and continued regular in that respect; she regained her flesh, color, and appetite; was able to take a long walk, had no bearing down, nor difficulty in passing water; and, when I saw her in January, 1841, she told me that she enjoyed better health than she had done for many years. Nothing solid had ever passed from the vagina since the operation.

Early in the year, symptoms of pregnancy manifested themselves; and I delivered her of a six months' fœtus, on July 7th, in the same year, 1841. Since then I have also attended her with four other children. On one occasion the placenta adhered, and I had to introduce my hand to remove it. I sought for a polypus in the cavity, but there was nothing like one. On onother occasion, after the placenta had been expelled naturally, she was harassed with violent spasmodic bearing down pains, which induced me to pass my hand into the uterus; I there found the fundus and posterior part of the body protruded considerably downward and forward, there existing evidently a disposition for inversion to occur again; this, however, was obviated by the introduction of the hand."

This ought to give us additional confidence in any effort we may undertake for the purpose of remedying, by manipulation, this serious accident.—*Med. Times and Gazette, November 5th*, 1859.

